# Case Report: Multicentric Reticulohistiocytosis Associated With Posterior Mediastinal Adenosquamous Carcinoma, Antinuclear Antibody Positivity and Lupus Anticoagulant Positivity

**DOI:** 10.3389/fimmu.2021.749669

**Published:** 2022-01-07

**Authors:** Ziyi Tang, Xiangpeng Wang, Zijing Xia, Zhongming Wang, Yi Zhao, Yi Liu

**Affiliations:** ^1^ Department of Rheumatology and Immunology, West China Hospital, Sichuan University, Chengdu, China; ^2^ Laboratory of Rheumatology and Immunology, West China Hospital, Sichuan University, Chengdu, China

**Keywords:** multicentric reticulohistiocytosis, mediastinal malignancy, paraneoplastic syndrome, rare disease, case report

## Abstract

Multicentric reticulohistiocytosis (MRH) is a rare systemic disease of non-Langerhans cell histiocytosis. A number of studies in the literature have documented that it can coexist with malignancy or autoimmune disease, making it difficult to determine the most appropriate therapy. Here, we present a case study of MRH associated with posterior mediastinal adenosquamous carcinoma along with antinuclear antibody positivity and lupus anticoagulant positivity. The patient experienced 6 months of clinical benefit after surgical resection and chemoradiotherapy of the mediastinal malignancy. This case adds to the available literature on multicentric reticulohistiocytosis associated with different types of malignancy and provides supplementary clinical data on the coexistence of this syndrome with malignancy and immune system abnormalities. To the best of our knowledge, this is the first case study describing MRH accompanied by posterior mediastinal adenosquamous carcinoma and lupus anticoagulant positivity. The unknown aetiology and polymorphic clinical presentation of MRH warrants further investigation.

## Introduction

Multicentric reticulohistiocytosis (MRH), also known as lipoid dermatoarthritis, is a rare systemic disease of non-Langerhans cell histiocytosis (N-LCH). The disease was first systematically reported in 1937 by Weber & Freudenthal, and there have been no more than 400 cases reported globally, mostly presenting in middle-aged white women (1, 2). The main manifestations of MRH are destructive polyarthritis and papulonodular skin lesions that occasionally affect other organs, including the lungs, heart, liver and kidneys (3). The aggressive erosion of the distal interphalangeal joints generally precedes the presentation of the skin phenotype and is the most frequent manifestation of MRH; thus, it can be used as the basis for differential diagnosis. Skin lesions most frequently appear on the hands and face, including flesh-coloured to pinkish papules and nodules. The appearance of “coral beads” around the nail folds is a characteristic sign of MRH. Dermatoscopy of these lesions were reported to show some typical features: a pattern with various shades of yellow/orange/reddish (like the “setting-sun” pattern), central white scar-like patches and streaks, brown reticular structures and linear teleangectasias, which may be a clue to diagnosis (3–6). MRH patient skin biopsies typically show a large degree of lymphohistiocytic proliferation and multinucleated giant cells with fine granules and granular ground glass-like cytoplasm. These features are used to establish a diagnosis of MRH by histology. Unfortunately, the molecular pathogenesis of MRH remains poorly understood. Alternatively, activated macrophages, elevated proinflammatory cytokines and osteoclast activation may be involved in the affected tissues (7, 8).

Some researchers hold the view that MRH is a neoplastic or paraneoplastic disease, and approximately 25% of MRH patients do indeed have an underlying malignancy (9). On the other hand, an analysis of the literature reveals that approximately 15%-29% of MRH cases coexist with autoimmune diseases such as Sjogren’s syndrome, rheumatoid arthritis, systemic lupus erythematosus, systemic sclerosis, dermatomyositis, and undifferentiated connective tissue disease (9, 10). Several cases of MRH-concomitant thrombosis have also been reported (9, 11–13). However, the relationship among MRH, malignancy, autoimmune disease, and thrombosis remains unclear because of the rarity of the disease. It therefore remains to be determined whether these are mere associations or truly part of the disease course. There is no appropriate treatment for MRH, and several therapeutic agents have been used with varying efficacy. Here, we describe the case of a patient who had posterior mediastinal adenosquamous carcinoma with positive antinuclear antibody (ANA) and lupus anticoagulant (LA) positivity; the patient experienced 6 months of clinical benefit after surgical resection and chemoradiotherapy for the mediastinal malignancy.

## Case Description

A previously healthy, 36-year-old Chinese man presented with papulonodules on his face and hands that had emerged over an 8-month period, along with experiencing polyarthritis for half a year. He reported smoking half a pack per day for 17 years. His family history was significant for malignancy in that his mother had breast cancer.

**Figure 1:**
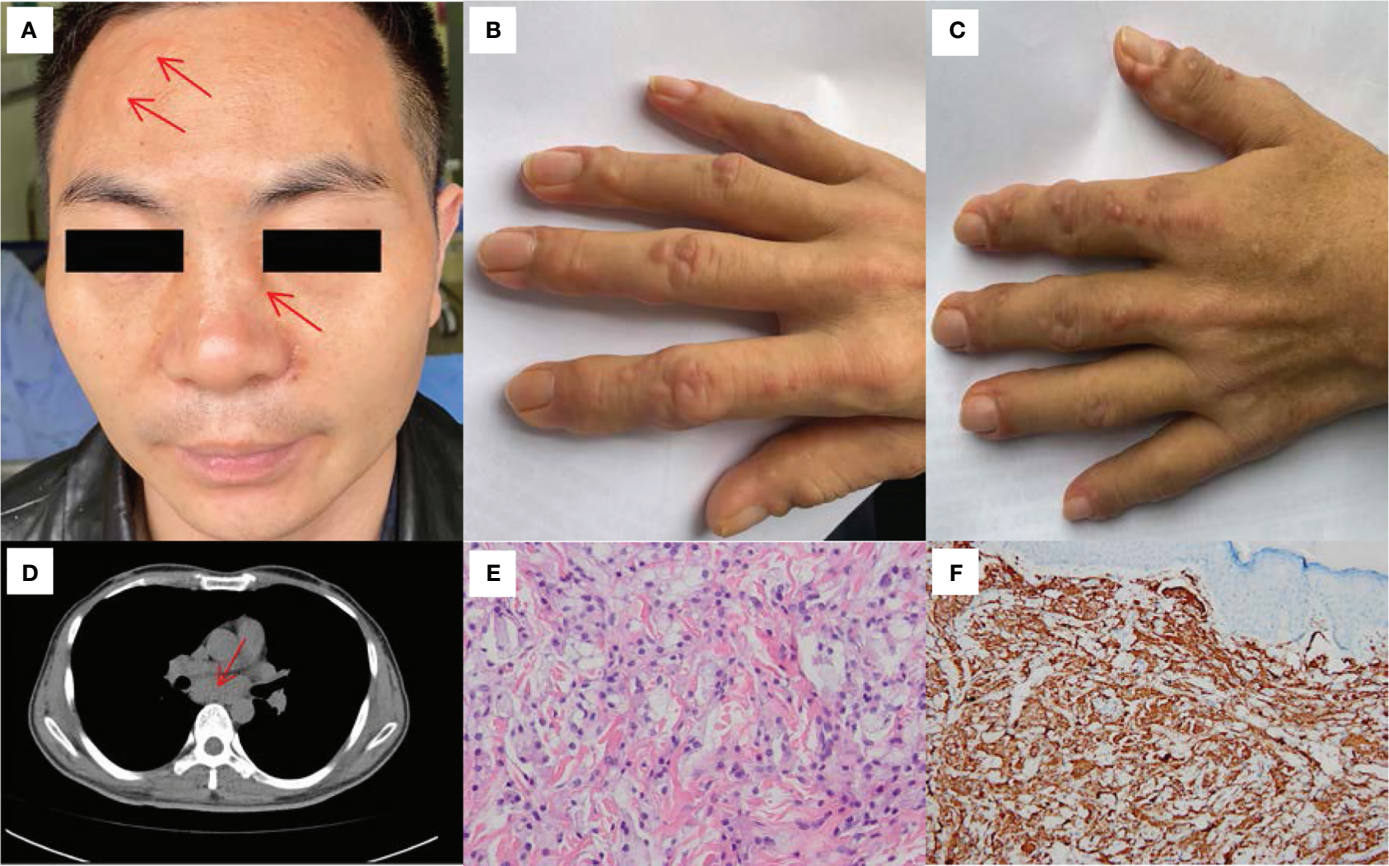
Clinical, HRCT and histological features of our patient. **(A)** Multiple flesh-colored to pinkish papules and nodules were noted on his face (before treatment). **(B)** Flesh-colored to pinkish papulonodular lesions were observed on the right hand (before treatment). **(C)** Flesh-colored to pinkish papulonodular lesions were observed on the left hand (before treatment). **(D)** HRCT of the chest revealed a mass of lymphoglandula within the posterior mediastinum. **(E)** Biopsy of papule skin lesions showed proliferation of abundant foamy macrophages and histocytes in the dermis (HE×400). **(F)** Dermal and epidermotropic foamy macrophages were strongly positive for CD68 (×200).

On admission, a physical examination revealed multiple flesh-coloured to pinkish papules and nodules on his face (**Figure 1A**), distal interphalangeal and proximal interphalangeal areas, and papulonodular lesions arranged around the nail plica in a typical “coral bead” pattern (**Figures 1B, C**). The cutaneous lesions were tender but without pruritus. There was also significant tenderness noted in the bilateral interphalangeal joints, wrists, elbows, shoulders, ankles, knees and hips, with no signs of swelling.

Laboratory tests showed slightly elevated inflammatory markers, including a C-reactive protein (CRP) level of 23.4 mg/L (reference range, <5 mg/L), erythrocyte sedimentation rate (ESR) of 49 mm/h (reference range, <21 mm/h), interleukin-6 (IL-6) level of 9.69 pg/ml (reference range, 0-7 pg/ml) and tumour necrosis factor (TNF) level of 9.26 pg/ml (reference range, <7 pg/ml). Routine blood, biochemistry, urinalysis, C3 and C4 levels were normal. The levels of tumour markers (CEA, AFP, CA125, CA19-9, CA72-4, CA15-3, CYFRA21-1 and PSA) did not exceed the reference range. Further analysis revealed that the patient showed ANA (titer 1:320), LA (1.93, reference range, 0.8-1.2, LA performed with dilute Russell’s viper venom time [dRVVT] screen/confirm), PPD test and tuberculosis interferon-γ release assay positivity. The level of fibrinogen was 5.47g/L (reference range, 2.0-4.0 g/L). Tests for anti-dsDNA, anti-Smith, anticentromere, anti-ribonucleoprotein, anti-Jo1, anti-neutrophil cytoplasmic antibodies, rheumatoid factor (RF), anti-cyclic citrullinated peptide antibodies (ACPA), anticardiolipin antibodies (IgG, IgM and IgA), anti-β2-glycoprotein I antibodies (IgG, IgM and IgA) and HbsAg were all negative. Radiographs of the joints revealed osteoporosis in both hands and wrists and a slightly narrowed space between the first and second metacarpophalangeal joints of both hands (**Supplementary Material 1**). High-resolution computed tomography (HRCT) of the chest revealed emphysema and an inconspicuous mass of lymphoglandula within the posterior mediastinum (**Figure 1D**). The patient underwent endobronchial ultrasound (EBUS) puncture biopsy of the mediastinal lymphoglandula to exclude cancer after consultation with the Department of Respiratory Medicine. Contrast-enhanced CT imaging of the whole abdomen revealed calcified plaques in the abdominal aorta and its branches, with mural thrombosis. The above indicators led to a presumptive diagnosis of undifferentiated connective tissue disease and possible antiphospholipid syndrome. On day 7, the histopathologic features of the papulonodular skin lesions showed proliferation of abundant foamy macrophages and the presence of histocytes (**Figure 1E**). These lesions contained CD68-positive cells (**Figure 1F**) that were negative for S100, CD1a, Langerin, and Factor VIII markers (**Supplementary Material 2**). Thus, a histological diagnosis of MRH was made.

The patient received methotrexate (15 mg once a week) accompanied by oral prednisone (10 mg twice a day) and was discharged after 20 days of treatment. He continued the above regimen after discharge according to his doctor’s instructions. Approximately one month later, he was admitted again for assessment, which indicated that the papulonodular lesions had shrunk and that the inflammatory cytokines and molecules described above had returned to normal levels. Nonetheless, the patient’s joint pain was not significantly relieved.

**Figure 2:**
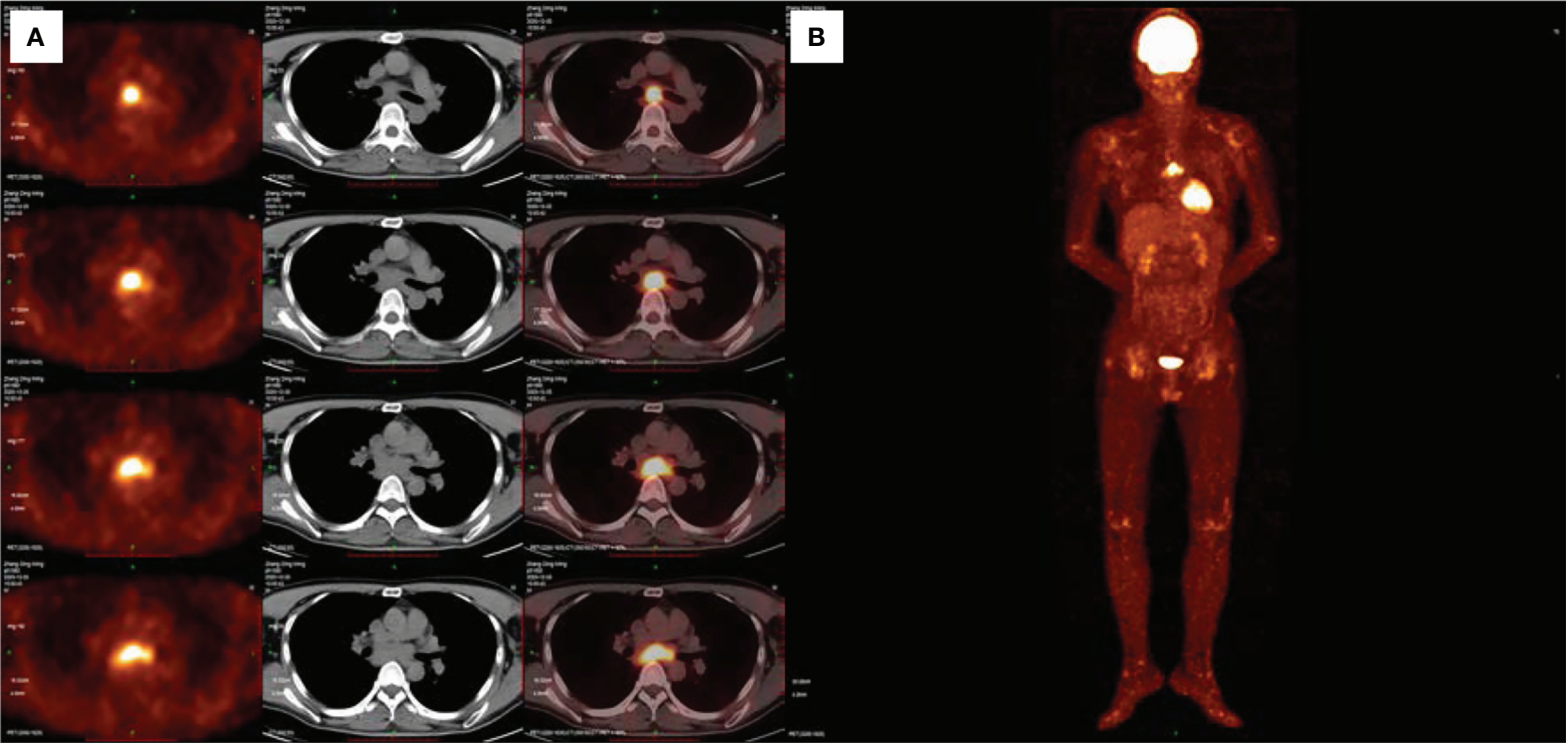
FDG-PET/CT showing increased glycometabolism in the mediastinal lymph nodes and soft tissue around the joints of the extremities **(A, B)**.

The results of EBUS puncture biopsy of the mediastinal lymphoglandula were available at the second hospital admission and showed a small number of abnormal cells (**Supplementary Material 3**). Immunohistochemistry showed cells positive for PCK, EMA, CK5/6 (focal), GATA-3, Ki67 and MIB-1 (20% positive). CR, TTF-1, P63, D2-40 and CgA were negative, and a diagnosis of epithelial neoplasia was made. To identify the primary lesion, fluorodeoxyglucose positron emission tomography/CT (FDG-PET/CT) was carried out, revealing increased glycometabolism in the soft tissue around the joints of the extremities and the mediastinal lymph nodes; the subcarinal lymph node findings confirmed the suspicion of malignancy (**Figure 2**). However, the primary tumour was not found despite exhaustive tests. The patient discontinued methotrexate and prednisone according to the recommendations of the multidisciplinary treatment (MDT) team.

To further clarify the diagnosis and treatment approach, the patient received video-assisted thoracoscopic surgery (VATS) to excise the mediastinal mass (6×3.5×2.8 cm) for genetic analysis. Malignant cells were identified in the background of abundant lymphoid tissue in the mediastinal mass (**Supplementary Material 4**). Histopathologic features of malignant cells were positive for P63 (focal), CK5/6 (focal), PCK, GATA-3, and Ki-67 (MIB-1, 50-60%) but not CR, PAX-8, CD5, CD117, WT-1, CgA, or EBER1/2, resulting in a diagnosis of poorly differentiated adenosquamous carcinoma. Genetic analyses revealed the presence of mutations in the SMARCA gene A4 (NM_003072.3:c.2438+1G>A IVS16, mutation rate 36.4%), amplification of EGFR, and a lack of PD-L1.

**Figure 3:**
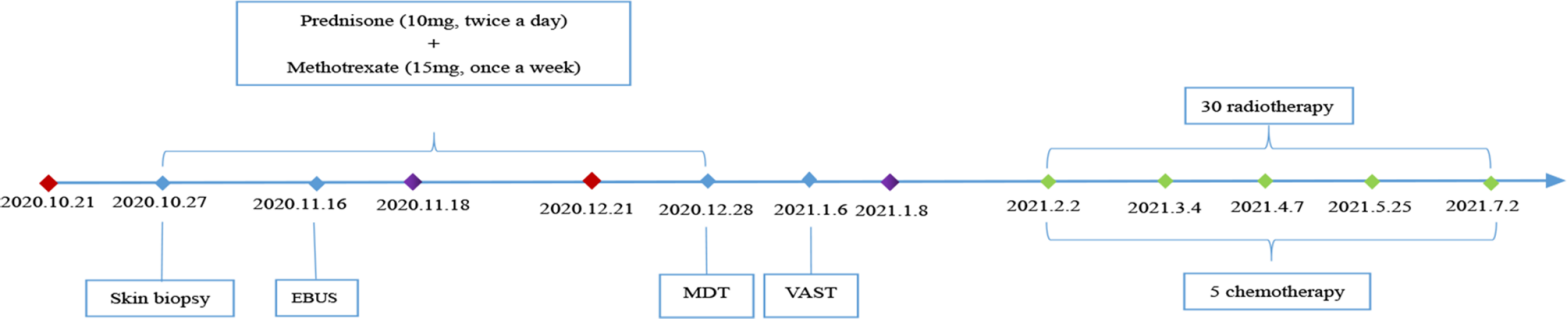
Clinical course of the patient. 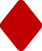 Date of admission; 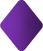 Date of discharge; 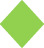 Date of chemotherapy; EBUS, Endobronchial ultrasound; MDT, Multidisciplinary treatment; VATS, Video-assisted thoracoscopic surgery.

After discussion with oncologists, chemoradiotherapy was administered, namely, intravenous paclitaxel 210 mg for 1 day combined with intravenous carboplatin 450 mg for 1 day (once every 3 weeks) and radiotherapy for the mediastinal lymph nodes. After 6 months of follow-up from the diagnosis of malignancy (5 cycles of chemotherapy and 30 cycles of radiotherapy), the patient’s joint pain was significantly reduced, and the papulonodular lesions had almost cleared, without any specific treatment. The latest re-examination on June 29, 2021, revealed that the patient was ANA negative and the LA level had decreased to 1.32, with no signs of tumour recurrence. The patient is still being followed-up. The changes to the lesions on the hands are presented in **Supplementary Material 5**. The patient’s clinical course is shown in **Figure 3**.

## Discussion

N-LCH consists of a group of rare diseases with different clinical presentations, pathogeneses and morphologies, including MRH, Erdheim-Chester disease, and Rosai-Dorfman disease, among others (14). MRH can be differentiated from Erdheim-Chester disease and Rosai-Dorfman disease by its pathological and clinical manifestations. In our patient, a diagnosis of MRH was made based on the typical skin presentation and skin lesion histology, but destructive arthritis was not observed at that time. This could be attributed to the very early initiation of treatment with disease-modifying drugs. Although inflammatory markers have been suggested to play a role in the development of disease, they seem to have had little special value for diagnosis; increased levels of cytokines have been reported in the literature (15), but the majority of cases have levels within the normal range.

The neoplastic and autoimmune nature of the disease has been a focus of previous MRH case reports since the first was published in 1937. MRH-related malignancies can involve various systems and organs, including the breast, bloodstream, gastrointestinal organs, gynaecological organs, lung, and skin. A previous study described an MRH patient presenting with two primary cancers, and metastatic occult cancer in the left axilla has also been reported (16, 17). Additionally, two cases of mediastinal tumours have been reported, both of which were thymomas of the anterior chest (18, 19). The present report of posterior mediastinal malignancy appears to be the first. Clinically, apart from primary thymic/thyroid masses and lymphomas, all other mediastinal masses can be considered rare tumours, and posterior mediastinal masses are generally neurogenic tumours, followed by sarcomas (20). This means that our case is extremely rare.

Another concern in our case is mutated *SMARCA4*. In the analysed literature, *SMARCA4* encodes one of the enzymatic (ATPase) subunits of the mammalian *SWI/SNF* family, a chromatin remodelling complex that directs nucleosomes and modulates gene expression. The gene is located on chromosome 19p13.2 and encodes the BRG1 protein, inactivating mutations of which result in its loss of expression and characterize a group of malignancies (21). Thus, we introduce a rare malignancy, called *SMARCA4*-deficient thoracic tumour (SMARCA4-DTT), a distinct entity of undifferentiated thoracic malignancy that was first described in 2015 (22), presenting aggressive behaviour with compressive or infiltrative features and conferring a poor prognosis. SMARCA4-DTT mainly involves the mediastinum, lung, and/or pleura and mostly occurs in heavy smokers with emphysema; it requires complete identification and differentiation from other epithelial malignancies involving the thoracic region, and fortunately, many physicians are becoming increasingly aware of this disease (23, 24). The majority of SMARCA4-DTTs have been reported as SMARCA4-deficient thoracic sarcomas thus far, but an increasing number of carcinomas have been reported (25). We were surprised to find that the malignancy presentation in our patient was highly similar to SMARCA4-DTT. There is currently no recognized diagnostic standard for SMARCA4-DTT, and our case may provide a reference for future research.

At present, whether MRH is a paraneoplastic syndrome remains controversial. We do find that some patients, such as the one described in this case, experience remission of the lesions after anti-tumour treatment, or the onset of MRH occurs just prior to relapse. Nevertheless, the fact that malignancies are observed in association with MRH and the fact that these two diseases do not always run a parallel course makes the paraneoplastic nature of this entity questionable. A Japanese study performed whole-exome sequencing and RNA sequencing in two patients with MRH, and the results indicated that MRH should be considered a neoplastic disease caused by activation of the RAS-MAPK pathway and that the effects of chemotherapy on this disease are promising (26).

Our patient experienced a very insidious onset of malignancy. Due to the background of negative tumour markers in routine serum tests and the lack of symptoms, it can be difficult to detect this type of malignancy by chest HRCT, especially by inexperienced clinicians. In recent years, the application of FDG-PET/CT in MRH has attracted increasing attention (27–29). FDG-PET/CT is a potentially useful way to detect and evaluate the grade of inflammatory involvement of MRH and to assess the possible associations with malignant neoplasms, as FDG is easily taken up by granulomas and inflammatory cells.

In case reports, MRH combined with immune diseases is common, and ANA, ACPA and RF positivity can be observed, but laboratory testing showing LA positivity is unusual. This is the first report of MRH in which ANA and LA were both found to be positive. Since our patient only had mural thrombosis in the abdominal aorta and its branches, no anticoagulants were administered. Due to the lack of clinical manifestations and other specific serological indicators, connective tissue disease could not be diagnosed in our case. After surgical resection and chemoradiotherapy for the malignancy, we observed that the ANA of the patient became negative and there was a significant decrease in LA. This reminded us to consider a positive result of ANA and LA in our case dialectically because studies have shown that ANA and LA are associated with malignancy (30–33).

Given that its clinical manifestations are similar to rheumatic conditions, it has been proposed that MRH is an autoimmune or inflammatory disease, so treatments such as the administration of corticosteroids, methotrexate, thalidomide (34), bisphosphonates, and biological anti-inflammatory agents [etanercept, adalimumab (35), infliximab, tofacitinib (36), upadacitinib (37)] have been employed with varying efficacy. Although our patient was sensitive to methotrexate treatment, we had to discontinue it because of the malignancy. The main focus of treatment was to prevent joint damage, but priority had to be given to the management of the malignancy once it had been diagnosed. It is worth noting that MRH can be associated with either immune disease or malignancy, making the selection of an appropriate therapeutic regimen challenging because the two possibilities are mutually exclusive. In our patient’s case, MRH remission was observed after treatment of the malignancy without any other specific treatment, although his long-term prognosis is still unknown.

In conclusion, although a definitive diagnosis of MRH rests upon histological examination of biopsy specimens, careful roentgenological interpretation and analysis of the clinical features are the keys to early diagnosis of this disease. The types of malignancies that can be complicated by MRH are diverse and lack specificity, and once diagnosed, patients with MRH warrant thorough examination. FDG-PET/CT should be recommended as an essential procedure to exclude underlying malignancy, regardless of whether tumour markers show negativity. At the same time, screening for immune system diseases and evaluation of the risk of thrombosis should not be neglected. Due to the high incidence of joint destruction, systemic involvement and malignancy, MRH needs to be recognized and given more clinical attention. The current study of this disease is insufficient; we believe that this case report will provide guidance to medical practitioners encountering similar clinical presentations.

## Data Availability Statement

The original contributions presented in the study are included in the article/**Supplementary Material**. Further inquiries can be directed to the corresponding author.

## Ethics Statement

The studies involving human participants were reviewed and approved by Sichuan University West China Hospital Health Research Ethics. Written informed consent for participation was not required for this study in accordance with the national legislation and the institutional requirements. Written informed consent was obtained from the individual(s) for the publication of any potentially identifiable images or data included in this article.

## Author Contributions

ZT, XW, and ZX co**l**lected clinical data, summarized the case, reviewed the literature, and drafted the manuscript. ZW and YZ provided the case. YL reviewed and summarized the case. All authors contributed to the article and approved the submitted version.

## Funding

This work was supported by 1·3·5 project for disciplines of excellence, West China Hospital, Sichuan University (Grant Number: ZYGD18015, ZYJC18003).

## Conflict of Interest

The authors declare that the research was conducted in the absence of any commercial or financial relationships that could be construed as a potential conflict of interest.

## Publisher’s Note

All claims expressed in this article are solely those of the authors and do not necessarily represent those of their affiliated organizations, or those of the publisher, the editors and the reviewers. Any product that may be evaluated in this article, or claim that may be made by its manufacturer, is not guaranteed or endorsed by the publisher.
