# Supplementary material for: Case Report: Multicentric Reticulohistiocytosis Associated With Posterior Mediastinal Adenosquamous Carcinoma, Antinuclear Antibody Positivity and Lupus Anticoagulant Positivity
Source: Front Immunol. 2022 Jan 7;12:749669. doi: 10.3389/fimmu.2021.749669 (PMC8777097; doi:10.3389/fimmu.2021.749669)
Supplement: Supplementary file 1 [file DataSheet_1.docx]

Supplementary Material

# Supplementary Figures

**
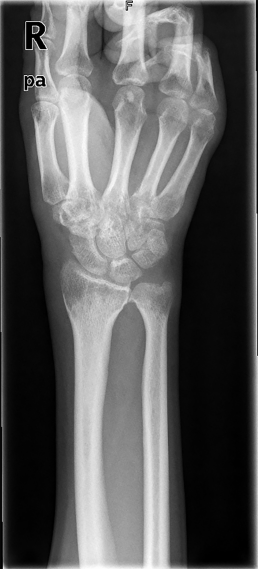

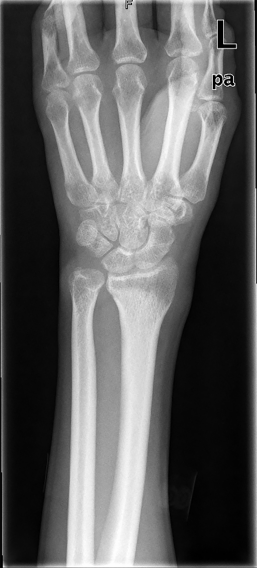

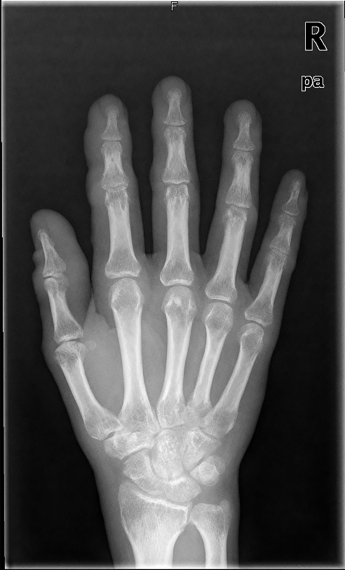

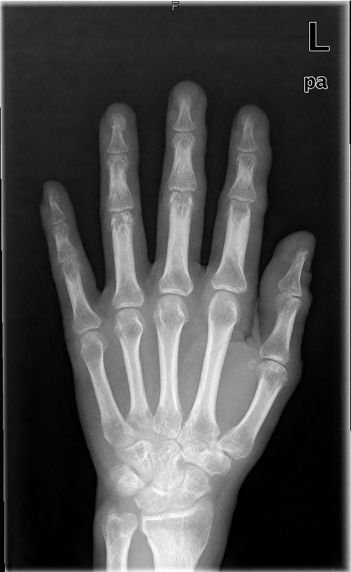
**

**Supplementary Material 1.** Radiographs of the joints revealing osteoporosis in both hands and wrists and a slightly narrowed space between the first and second metacarpophalangeal joints of both hands.


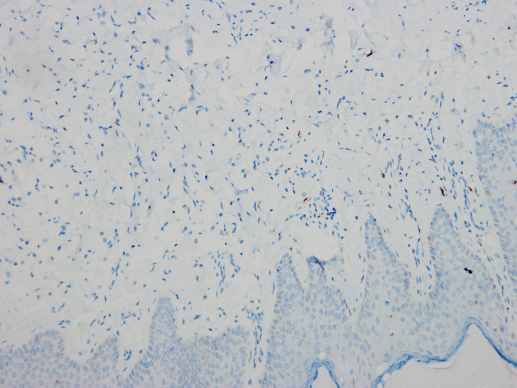


**D**

**C**

**B**

**A**


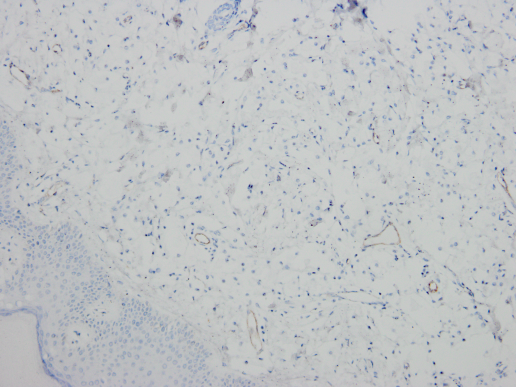

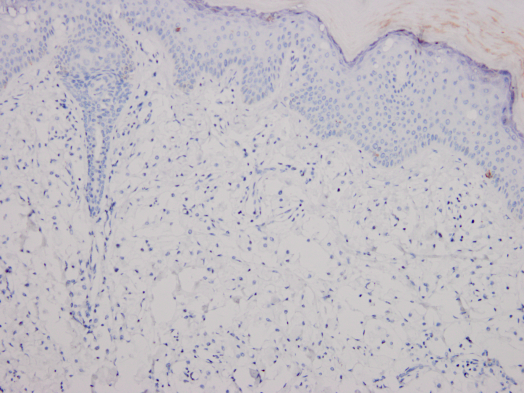


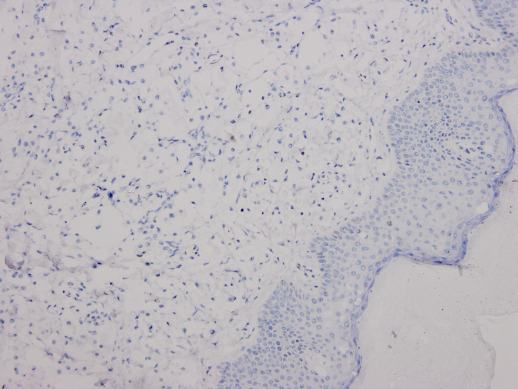


**Supplementary Material 2.** Immunohistochemistry of dermal lesions showing negative results for CD1a (**A**), Factor Ⅷ (**B**), S100 (**C**) and Langerin (**D**) markers (×200).


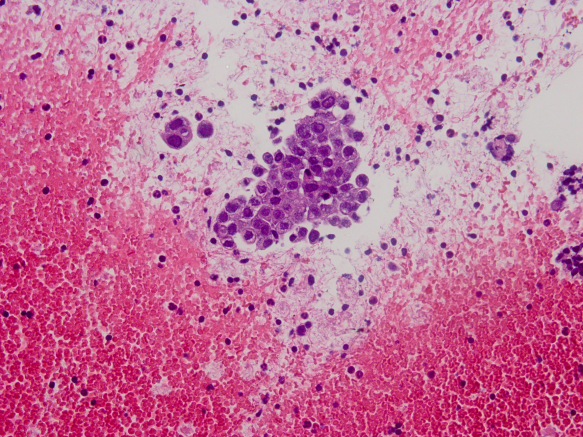


**Supplementary Material 3.** EBUS puncture biopsy of the mediastinal lymphoglandula showing a small number of abnormal cells (HE×400).


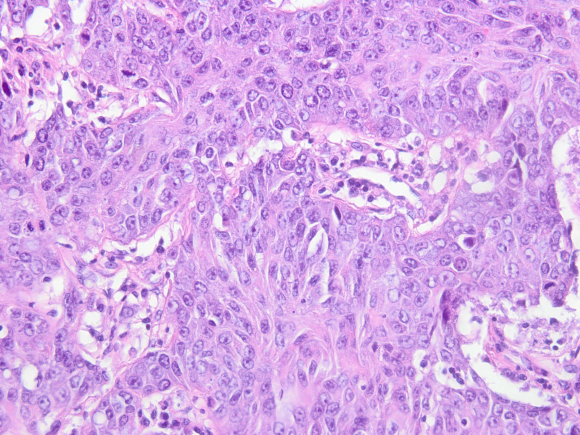


**Supplementary Material 4.** Malignant cells were identified in the background of abundant lymphoid tissue in the mediastinal mass. (HE×400).


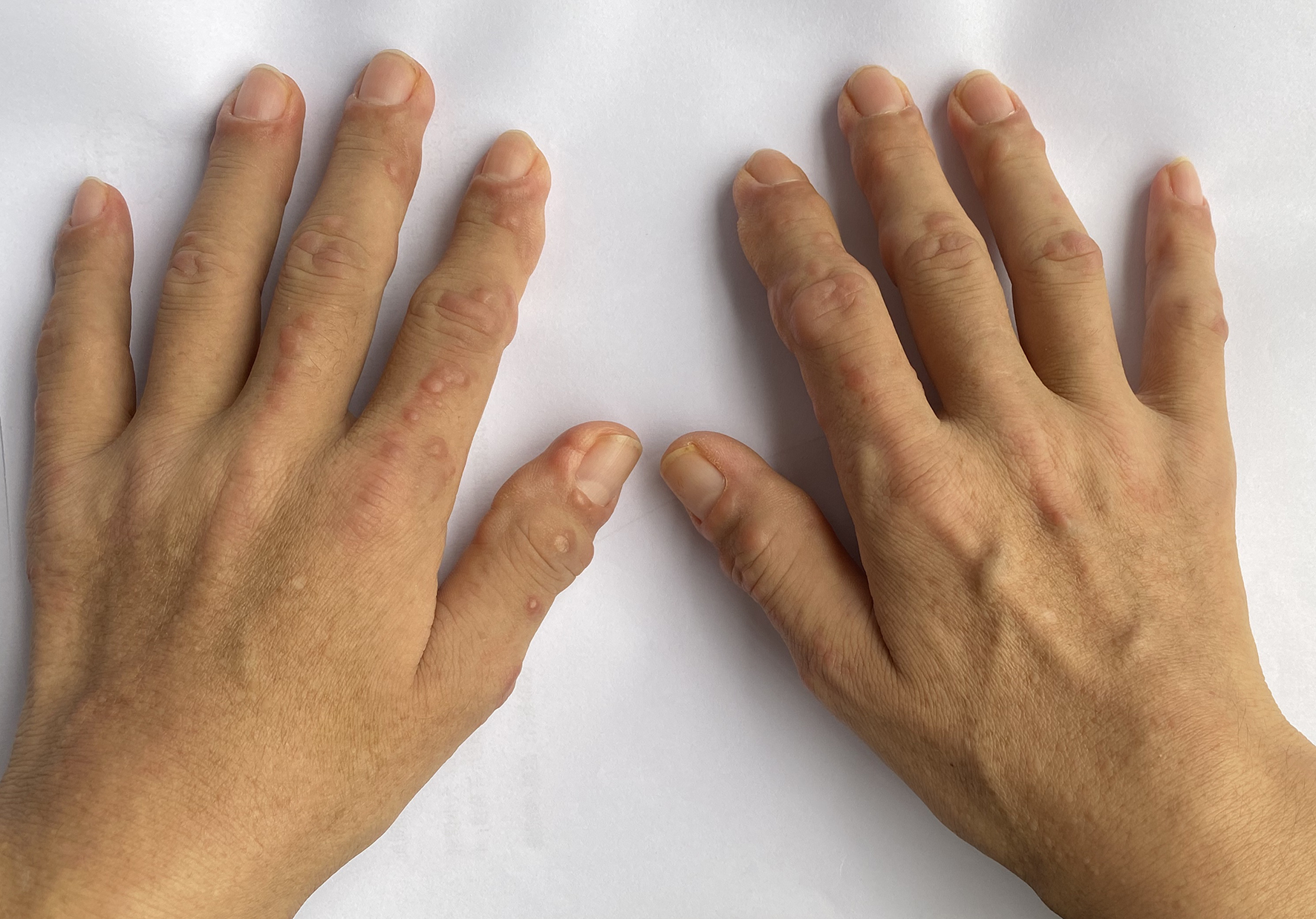


**A**


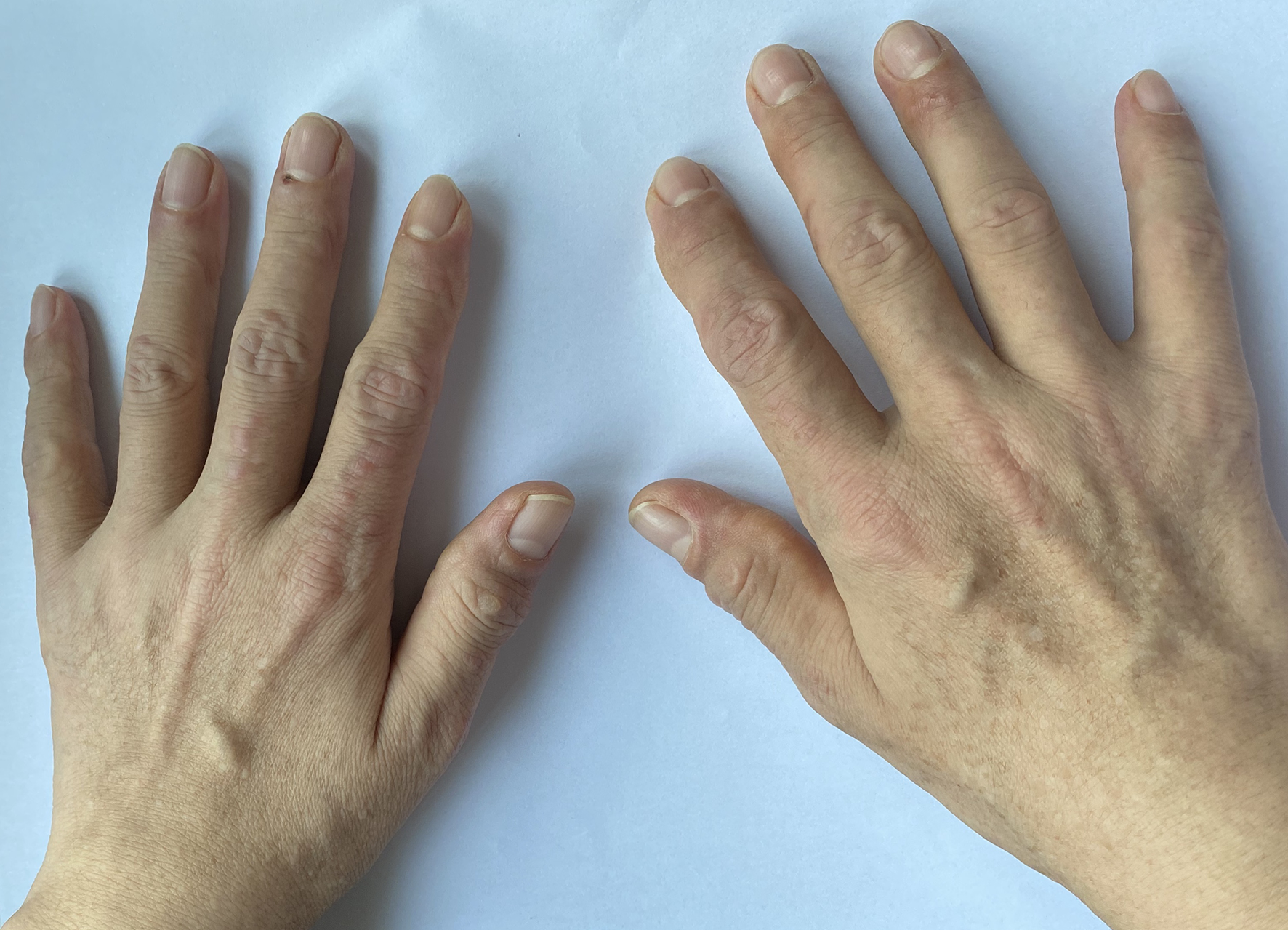


**B**

**
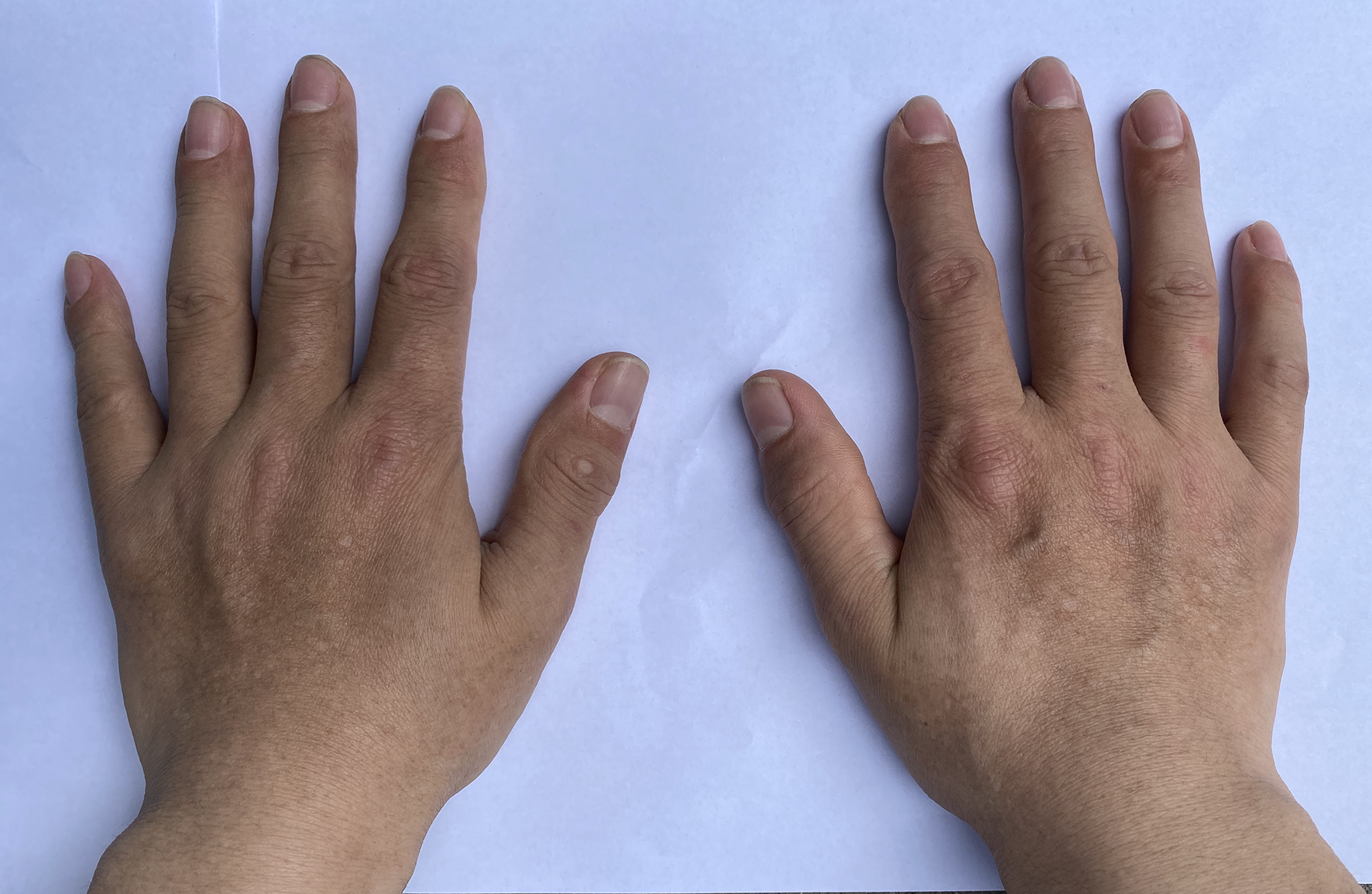
**

**C**

**Supplementary Material 5.** Papulonodular lesion changes.

(**A**) Papulonodular lesions on the hands before treatment.

(**B**) The papulonodular lesions were observed to shrink after treatment with methotrexate and prednisone.

(**C**) The papulonodular lesions had almost resolved after surgical resection of the malignancy and chemoradiotherapy.
