# Supplementary figures and images for: Case Report: Multicentric Reticulohistiocytosis Associated With Posterior Mediastinal Adenosquamous Carcinoma, Antinuclear Antibody Positivity and Lupus Anticoagulant Positivity
Source: Front Immunol. 2022 Jan 7;12:749669. doi: 10.3389/fimmu.2021.749669 (PMC8777097; doi:10.3389/fimmu.2021.749669)

Supplementary File


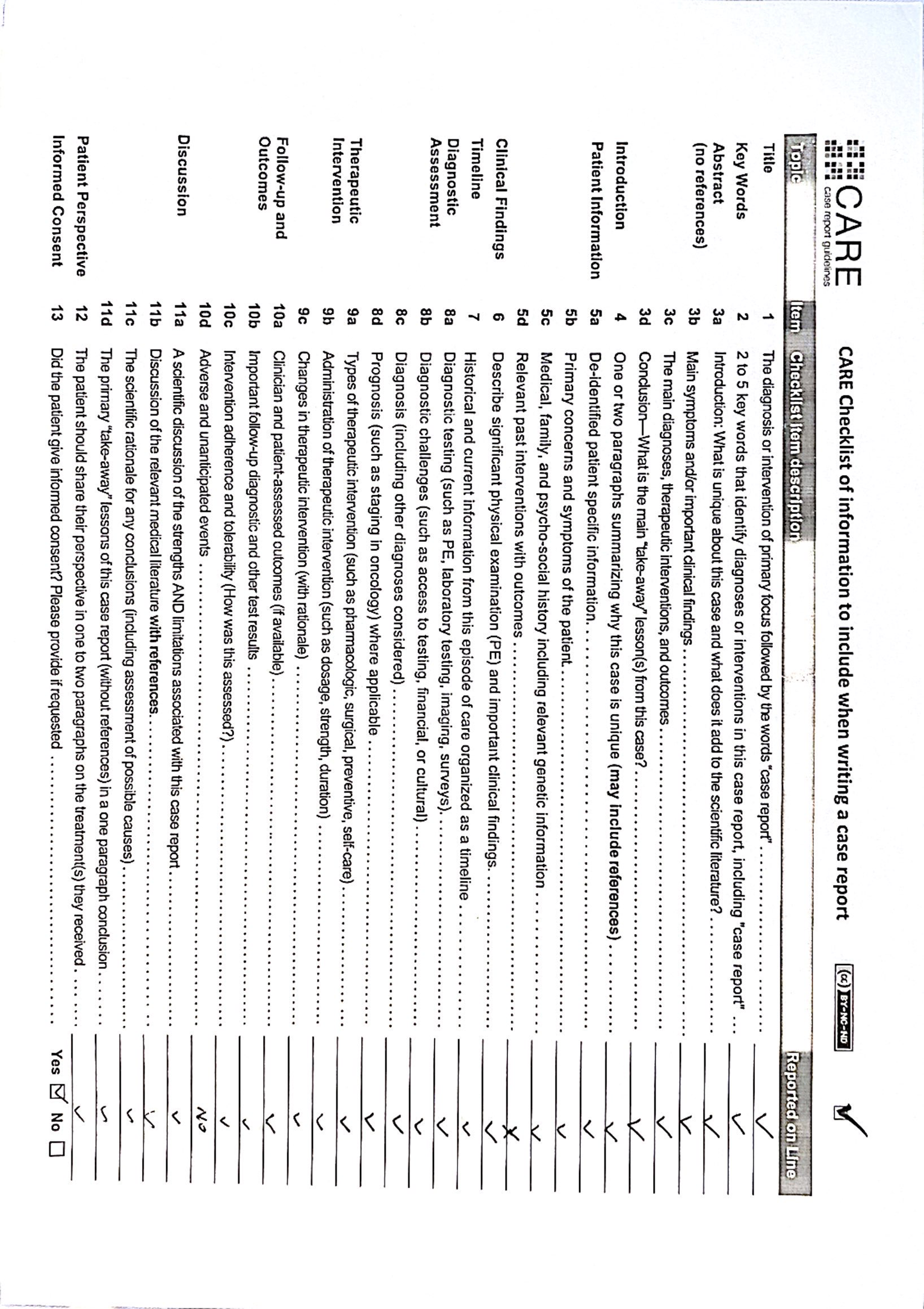


**Supplementary file**  CARE checklist

Supplement: Supplementary file 2 [file DataSheet_2.docx]
